# Machine Perfusion of the Liver: A Review of Clinical Trials

**DOI:** 10.3389/fsurg.2021.625394

**Published:** 2021-03-26

**Authors:** Nikolaos Serifis, Rudy Matheson, Daniel Cloonan, Charles G. Rickert, James F. Markmann, Taylor M. Coe

**Affiliations:** Center for Transplantation Sciences, Massachusetts General Hospital, Boston, MA, United States

**Keywords:** machine liver perfusion, normothermic liver perfusion, hypothermic liver perfusion, liver perfusion clinical trials, machine perfusion clinical trials

## Abstract

Although efforts have been made by transplant centers to increase the pool of available livers by extending the criteria of liver acceptance, this practice creates risks for recipients that include primary non-function of the graft, early allograft dysfunction and post-operative complications. Donor liver machine perfusion (MP) is a promising novel strategy that not only decreases cold ischemia time, but also serves as a method of assessing the viability of the graft. In this review, we summarize the data from liver machine perfusion clinical trials and discuss the various techniques available to date as well as future applications of machine perfusion. A variety of approaches have been reported including hypothermic machine perfusion (HMP) and normothermic machine perfusion (NMP); the advantages and disadvantages of each are just now beginning to be resolved. Important in this effort is developing markers of viability with lactate being the most predictive of graft functionality. The advent of machine perfusion has also permitted completely ischemia free transplantation by utilization of *in situ* NMP showed promising results. Animal studies that focus on defatting steatotic livers via NMP as well as groups that work on regenerating liver tissue *ex vivo* via MP. The broad incorporation of machine perfusion into routine clinical practice seems incredible.

## Introduction

Liver transplantation (LT) is the only curative treatment for patients with end-stage liver disease and select liver cancers. Significant advances over the last three decades in procurement, preservation, operative technique, and post-transplant immunosuppression have enabled liver transplantation to be an effective therapeutic option throughout the United States and much of the world. However, despite these advances, mortality on the waiting list remains high due to an ever-increasing shortage of suitable donor organs (1). Extended criteria donors (ECD), live donor liver transplant, expanded organ sharing, and a variety of organ preservation techniques have been introduced as means of increasing the pool of transplantable livers and decreasing waitlist mortality. Still, there remains an 8.4% of donated livers that are recovered and not used (2). A paradigm shift is needed. In this review, we explore the opportunities provided by these innovative and enabling technologies by review of the clinical trials that have been conducted to date as well as the prospects for novel applications in the future.

## Machine Perfusion

MP has been explored at a range of temperatures, with hypothermic machine perfusion (HMP) (Table 1) and normothermic machine perfusion (NMP) (Table 2) being the most commonly studied clinically. HMP can been performed with or without active oxygenation (HOP), as the low temperature (4–8°C) is sufficient to slow organ metabolism to limit oxygen demand (3). HOP aims to attenuate IRI by actively resuscitating the mitochondria, and restoring cellular energy stores to decrease release of reactive oxygen species post-reperfusion (4). On the other hand, NMP mimics normal physiologic conditions by perfusing the organ with oxygenated human blood or other oxygen carriers at or near 37°C. At this temperature, oxygenation is required to support metabolic demand. Since the liver is being perfused with oxygenated blood, normal cellular metabolism continues, allowing for the evaluation of organ health and function.

**Table 1:** HMP clinical trials and studies.

**Research group**	**Reference #**	**Year of publication**	**Study type**	**Method**		**Results**
				**Control group (# of recipients, donor type, preservation type)**	**Treatment group (# of recipients, donor type, preservation type)**	
Guarrera et al.	(20)	2010	Prospective cohort pilot study	20/DBD/SCS	20/DBD/HMP	Lower LFTs, Tbili, decreased hospitalization period in treatment group
Guarrera et al.	(20)	2010	Non-randomized clinical trial	3/DBD/SCS	3/DBD/HMP	Decreased expression of cytokines
Henry et al.	(21)	2012	Non-randomized clinical trial	15/DBD/SCS	18/DBD/HMP	Less up-regulation of inflammatory markers and adhesion molecules in treatment group. Less macrophage activation in histological analysis
Dutkowski et al.	(22)	2015	Randomized clinical trial	50/DCD/SCS	25/DCD/HOPE	Lower LFTs in treatment group
Guarrera et al.	(23)	2015	Non-randomized clinical trial	30/ECD/SCS	31/ECD/HMP	Fewer biliary complications, decreased hospitalization period in treatment group
Van Rijn et al.	(14)	2017	Non-randomized clinical trial	20/DBD/SCS	10/DCD/DHOPE	11 times increase in ATP, lower LFTs, TBili at 30 days post-transplantation in treatment group
Van Rijn et al.	(24)	2018	Randomized clinical trial	20/DBD/SCS	10/DCD/DHOPE	Fewer biliary complications and less biliary histologic damage in treatment group
Schlegel et al.	(25)	2018	Retrospective cohort study	100/50 DCD + 50 DBD/SCS	50/DCD/HOPE	Lower lactate and INR levels, less FFP, significantly higher rate of 5 year survival in treatment group
Burlage et al.	(26)	2018	Non-randomized clinical trial	9/DCD/SCS	10/DCD/DHOPE	DHOPE treated livers had a smaller increase in K+ levels and decreased needs in noradrenaline
Van Leeuwen et al.	(27)	2019	Non-randomized clinical trial	60/36 DBD + 24 DCD/SCS	11/DCD/DHOPE-COR-NMP	No statistically significant difference in patients survival at 3, 6, and 12 months post-transplantation between the three groups

**Table 2:** NMP clinical trials and studies.

**Research group**	**Reference #**	**Year of publication**	**Study type**	**Method**		**Results**
				**Control group (# of recipients, donor type, preservation type)**	**Treatment group (# of recipients, donor type, preservation type)**	
Op Den Dries et al.	(30)	2013	Pre-clinical study	No control group	4/DCD/NMP	Stable LFT, gradual improvement of bile production
Ravikumar et al.	(31)	2016	Non-randomized clinical trial	40/32 DBD + 8 DCD/SCS	20/16 DBD + 4 DCD/NMP	DCD control livers reached significantly higher levels of AST
Angelico et al.	(32)	2016	Retrospective cohort study	12/8 DBD + 4 DCD/SCS	6/4 DBD + 2 DCD/NMP	NMP treated livers required less vasopressors to reach hemodynamic stability
Nasralla et al.	(33)	2017	Randomized clinical trial	101/80 DBD + 21 DCD/SCS	121/87 DBD + 34 DCD/NMP	PRS was significantly more common in SCS group, 10% of NMP group vs. 30% of SCS group developed EAD
Watson et al.	(34)	2017	Case series	No control group	12/3 DBD + 9 DCD/NMP (6 hyperoxic + 6 normoxic)	Significantly less peak ALT in normoxic group at post-transplantation day 7, significantly less PRS, and vasoplegia in normoxic group
Watson et al.	(35)	2018	Case series	No control group	22/6 DBD + 16 DCD/NMP	Correlation between ALT in perfusate at 2 h and peak ALT at post-transplantation day 7
Mergental et al.	(36)	2018	Pre-clinical study	No control group	12/4 DBD + 8 DCD/NMP	Non-LC livers had macrovesicular steatosis, biliary cells damage and also mitochondrial injury, lactate-clearing livers produced more bile than the non-lactate-clearing livers
Ceresa et al.	(37)	2019	Non-randomized clinical trial	104/73 DBD + 31 DCD/Continuous NMP	30/23 DBD + 7 DCD/pSCS-NMP	Significant reduction in perfusate glucose and lactate in treatment group. Significantly higher hepatic artery flow rates at the beginning of perfusion compared to control group
Bral et al.	(38)	2019	Non-randomized clinical trial	17/13 DBD + 4 DCD/local NMP	26/20 DBD + 6 DCD/back-to-base NMP	The recipients of treatment group had a shorter ICU and hospital stay, 100% 30-day patient and graft survival for both groups. Six month survival rate was 100 and 88% for back-to-base and local NMP groups, respectively
Matton et al.	(39)	2019	Pre-clinical study	No control group	23/5 DBD + 18 DCD/NMP	Correlation between biliary pH, glucose, and bicarbonate concentrations in perfusate and BDI score. The higher the value of these markers, the lower the BDI score
Ghinolfi et al.	(40)	2019	Randomized clinical trial	10/DBD/SCS	10/DBD/NMP	Histologically more severe damage in SCS preserved livers 2 h post-reperfusion than in NMP treated livers, correlation between recipients' lactate levels and perfusate concentrations of IL-6, IL-10, and TNF-a
Jassem et al.	(41)	2019	Non-randomized clinical trial	27/DBD/SCS	12/DBD/NMP	The hospitalization period correlated positively with the frequency of IL-17 producing CD4+ T cells, 306 genes related to tissue regeneration and platelet function were expressed in NMP group, while 256 genes related to immune response and platelet function were expressed in the SCS group
Watson et al.	(42)	2019	Retrospective cohort study	187/DCD/non-NRP	43/DCD/NRP	Early allograft dysfunction incidence was significantly lower in NRP group, lower peak ALT in the treatment group in the first post-op week, non-NRP group had more biliary complications. None of the NRP treated livers developed cholangiopathy, while 27% of the non-NRP livers did
Mergental et al.	(43)	2020	Non-randomized clinical trial	44/24 DBD + 20 DCD/SCS	22/12 DBD + 10 DCD/NMP	No significant difference in 1 year survival between the two groups, anastomotic and NAS in treatment group
Matton et al.	(44)	2020	Pre-clinical study	No control group	12/3 DBD + 9 DCD/NMP	HDmiR122 and CDmiR122 correlated positively with peak AST levels at the end of the perfusion, Perfusate HDmiR122/CDmiR122 ration after 30 min of NMP correlated positively with the Suzuki score at the end of the perfusion

## HMP Principles

Until just recently, the field has relied almost exclusively on SCS for preservation of livers for transplant. However, cellular energy reserve depletion, change in cytosolic ion concentrations, instability of cellular membranes and increased vulnerability of liver endothelial cells to ischemia/reperfusion injury are deleterious alterations during cold storage (5). SCS has failed to meet the modern needs for organ preservation as we move to increasingly broad organ sharing resulting increased cold ischemia time and the expanding use of marginal grafts.

HMP has been tested both in preclinical animal models and clinical trials as a theoretically superior preservation method to SCS. In 1960, Folkert Belzer developed the first HOP machine (6), which in 1968 is used to perform the first HOP preserved human kidney transplant (7). The need for customized resources for that perfusion system along with the introduction of UW solution by Belzer in 1987, resulted in SCS being favored over HMP for clinical practice. This was reversed in the 1990s, when ECD kidney usage increased and technological advancements allowed for improvements in machine perfusion technology.

At low temperature, oxidative energy [i.e., adenosine triphosphate (ATP)] is generated by electron transport system in the mitochondria in reduced rate, which leads to development of HOP. HOP requires maintained temperature between 4 and 8°C with metabolic substrate enriched perfusate to the allograft. This condition promotes ATP generation, and replenish allograft's energy reserves (8). During ischemia, lack of nutrient and oxygen shifted cellular respiration from aerobic to anaerobic respiration. Several studies indicated that HOP provided three different protective mechanism to allograft during preservation. Firstly, provoke up-regulation of Kruppel-like factor 2 (KLF2) and endothelial nitric oxide synthase (eNOS) which provide cellular protection to endothelial cells from ischemic insult (5). Secondly, reduced damage associated molecular patterns (DAMPs), released by hepatocyte during early reperfusion of donation after circulatory death (DCD) livers (6, 9). Finally, low temperature oxygen rich perfusion allows intracellular ATP regeneration, limits reactive oxygen species (ROS) accumulation in the cell, and reduction of lactate production (8). HOP is reported to be cost efficient because it provides perfusionist flexibility to employ UW solution or hemoglobin-based oxygen carrying solution (i.e., Hemopure, etc.) as perfusate (7).

## NMP Principles

NMP seeks to recapitulate physiologic conditions by perfusing the organ at 34–38°C with oxygenated blood, nutrients, and medications. It was first clinically applied in 1984 to preserve heart and lung grafts during distant procurement (10).

In 2007, Steen et al. first used NMP as a method to assess viability in deceased donor lungs that were rejected for transplantation. After 17 h of *ex vivo* perfusion of the lung, it was successfully transplanted to a patient that experienced normal post-operative course (11).

Compared to HMP, NMP facilitates normal aerobic metabolism of the graft, which allows assessment of its viability. This becomes an important tool in selecting livers that are likely to have better outcomes post-operatively, especially when it involves marginal grafts. Pre-transplantation viability assessment of the livers will potentially reduce the incidence of post-LT complications. NMP can also be employed to extend periods of storage, as recently demonstrated by Eshmuminov et al. (12) who perfused human livers for 5–7 days. This could help facilitate long-distance transportation, thus optimizing graft-recipient matching. Another major advantage of NMP is the ability to pretreat the organ with medications *ex vivo*. This may avoid systemic adverse effects in the recipient and also provides the transplant team with the ability to assess the organ's response to various pharmacologic interventions (13). With NMP there is a theoretical danger of warm ischemic injury to the organ, in the event of a device technical failure. Fortunately, to date this has not yet been reported.

## Alternatives to Blood Products

Adequate oxygenation of the organ, via an oxygen carrier in perfusate, is required for NMP (14). While blood-based perfusate has typically been used for NMP, it is accompanied by the theoretical risk of immune-mediated responses, red blood cell (RBC) hemolysis, thrombus formation, and a risk of blood-borne infectious transmission (14). The use of human blood products is costly and can be logistically challenging if not provided by the donor hospital. It is also possible to avoid RBC altogether, with acellular oxygen carriers that have similar oxygen carrying capacity to human hemoglobin (15–17).

In 2018, Matton et al. (18) explored alternative solutions for liver NMP, where three groups with different perfusates were compared. The first group used FFP and pRBCs as perfusate (*n* = 12). The second group used HBOC-201 (Hemopure), a solution based on polymerized bovine hemoglobin-based oxygen carrier, and FFPs (*n* = 6). Finally, livers (*n* = 6) were perfused with Hemopure and gelofusine, a gelatin-based colloid solution. Results showed that during NMP, both liver ATP was higher for both Hemopure groups, while ALT was lower. Two hours post-perfusion, livers in the Hemopure group produced more bile than those in the RBC+FFP group, but there were no significant differences between the two Hemopure groups. Additionally, lactate was cleared faster in the Hemopure group livers than those in the RBC + FFP group. Although the differences did not reach significance, these data may suggest that the Hemopure-perfused livers experience better aerobic metabolism than the RBC + FFP perfused livers. This approach using Hemopure, a bovine hemoglobin multimer, was recently applied successfully in a series of liver transplants (19). If continued success with this off-the-shelf oxygen carrier is observed, it will simplify the perfusion process and potentially limit costs.

## HMP Clinical Trials and Studies

### HMP Impact on Liver Function Tests

In 2010, HMP clinical trial was pioneered by Guarrera et al. (20) where they conducted 20 liver transplantations using livers that underwent non-oxygenated HMP via both the hepatic artery and the portal vein. Despite it was not a randomized trial, patients were retrospectively matched to a control group of 20 liver recipients who received livers that underwent SCS with UW solution as perfusate. The trial results showed decreased hospitalization period as well as lower peak patient serum aspartate aminotransferase (AST), total bilirubin (TBili), and serum creatinine (SCr) in HMP group. It is important to note that there was no significant increase of these markers up to 14 days post-transplantation. This trial also showed a significant correlation between peak recipient ALT levels and both effluent ALT and effluent lactate dehydrogenase (LDH), adding a potential means of assessing liver function post-operatively. ATP levels were not measured in this series and active oxygenation of the circuit was not included though it is possible that low level oxygen delivery still occurred via the perfusate.

In 2015, Dutkowski et al. (22) published an international study comparing 25 DCD livers that underwent end-ischemic hypothermic oxygenated machine perfusion solely via portal vein (HOPE) and subsequent transplantation at their center to 50 DCD liver transplantations after normal cold storage from two centers abroad. Consistent with the studies already discussed, HOPE-treated livers manifested less liver enzyme release after reperfusion compared to non-perfused livers. While encouraging and provocative, these non-randomized findings are vulnerable to center and patient selection bias and will need to be confirmed in controlled randomized trials.

van Rijn et al. (14) conducted a study from 10 orthotopic liver transplantation that were perfused using end ischemic dual portal vein and hepatic artery hypothermic oxygenated machine perfusion (DHOPE) method. In DHOPE method, liver was procured from DCD donor and stored using cold preservative solution prior to being delivered to the recipient. Upon arrival, liver graft was oxygenated by the machine and perfused with 4 L of UW supplemented with 3 mmol/L glutathione. This data was matched to a control group that included 20 patients, who received livers after standard SCS. Results of this trial indicated that patients whose liver graft perfused with DHOPE method have lower serum ALT, γ-glutamyl transferase (GGT), alkaline phosphatase (ALP), and bilirubin at post-transplantation day 30 in comparison with patient whose liver graft perfused with SCS method. Furthermore, DHOPE method result indicated increase of intrahepatic ATP concentration by 11-fold due to oxygen availability from the perfusion machine and low temperature, which decreased ATP metabolism into ADP, production of DAMP, and ROS. This result suggested active oxygenation, characterized by low ALT and high concentration of intracellular ATP, during perfusion attenuated IRI.

### Molecular Analysis

Reperfusion of the liver at the time of transplant with warm, oxygenated blood releases cytokines, chemokines, and various compounds accumulated in the liver during cold preservation. These molecules provoke a complex inflammatory cascade that leads to parenchymal and sinusoidal endothelial cell injury (28).

In 2010, Guarrera et al. (29) compared three patients who received HMP livers to three patients who received SCS livers. Post-reperfusion biopsy samples of the SCS group showed significant up-regulation of ICAM-1, IL-8, and TNF-a compared with samples taken before liver transplantation. Donor grafts that underwent HMP had significantly attenuated up-regulation of these markers. This ultimately led to decrease of IRI and improved early function of the graft. Marginal livers from steatotic, elderly or DCD donors are the ones that will benefit the most of these findings.

Two years later, the same group expanded their work studying 18 HMP liver transplantations compared to 15 preserved with cold storage (21). They focused on measuring various markers during three stages of transplantation: at time of donation, immediately post-storage and after reperfusion phase. The level of expression of inflammatory markers, such as IL-1b and TNF-a, was significantly upregulated in the SCS group during the reperfusion phase compared to pre transplantation phase. On the other hand, there was less up-regulation in the HMP group during reperfusion. Many adhesion molecules (CCL21, CXCL1, P-Selectin, and MCP-1) were also found to be significantly upregulated at the post-storage phase in the SCS group. At the reperfusion biopsy, the aforementioned markers plus CXCL14, ICAM-1, and SDF-1a, were all significantly upregulated in the SCS group compared to the HMP group. CRP was upregulated in SCS at post-storage and reperfusion phases, while apoptotic proteins, such as cytochrome C and Caspase 3, were increased in the post-storage phase in the SCS group. Immunofluorescence labeling showed fewer CD68 expressing cells in the HMP group, especially in the reperfusion phase, indicating an attenuated macrophage activation. These results reveal the impact of HMP on a cellular level in reducing the oxidative stress, hypothermic shock, and ischemia-reperfusion injury that liver allografts would otherwise suffer in SCS.

### Biliary Complications

Perhaps the most feared and problematic complication after DCD liver transplantation is ischemic cholangiopathy. Up to 30% of DCD liver recipients developed non-anastomotic biliary stricture (NAS) (14).

In the non-randomized trial of Guarrera et al. (23), non-oxygenated HMP (*n* = 31) and SCS (*n* = 30) methods were compared and results indicated that 4 (13%) post-transplant biliary complications occurred in HMP group in comparison with 13 (43%) in control group. More specifically, there were three non-anastomotic biliary strictures (10%) in HMP vs. 10 (33%) in control group. This result positively affected liver recipient's hospitalization period, where patients whose liver grafts were perfused using non-oxygenated HMP were discharged earlier than those in the SCS group.

Study by van Rijn et al. (14, 24) with DHOPE method, showed lower incidence of bile duct damage in patient whose liver graft were perfused using DHOPE method compared to those in the SCS group. More specifically, SCS preserved livers showed a significant increase in the degree of mural stroma necrosis and extent of deep peribiliary glands (PBG) injury. Histological section of bile duct biopsy indicated that there is no significant bile duct lesion and cell death in the periluminal space and (PBG) injury post-reperfusion in DHOPE liver recipient. It is also noteworthy that patients whose liver was perfused using DHOPE method did not require re-transplantation due to NAS, while 20% of patients with SCS perfused liver require re-tranplantation due to NAS ~9 months after the initial transplantation (24).

In 2018, Schlegel et al. (25) conducted a study with 50 human DCD livers, that were transplanted at University Hospital in Zurich after a period of SCS following recovery and transport back to the transplanting center for HOPE treatment. These patients were matched to 50 DCD and 50 donors after brain death (DBD) untreated livers from UK. There were some significant differences in patient characteristics between the two centers that confound rigorous comparison. More specifically, donors in the HOPE group were significantly older than the donors of the control group and more than 40% of all donors were older than 60 years. Donor warm ischemia times were significantly longer in Zurich compared to UK. Livers in HOPE group were found to be micro- and macro-steatotic more frequently than the control ones. In addition, cancer patients received a DCD liver more often in the Zurich group than in the UK group. Despite these differences, results showed that patients undergoing LT without HOPE required significantly more fresh frozen plasma (FFP) transfusions during transplantation. HOPE improved clearance of lactate and liver synthetic function early post-transplantation, as demonstrated by lower lactate levels by the end of the operation, and lower INR at post-op day 1. Additionally, 14 (28%) recipients of untreated DCD livers developed acute graft rejection, as opposed to 2 (4%) recipients of HOPE treated livers. The number of non-anastomotic biliary strictures in untreated DCD livers was more than two times greater than the HOPE-treated livers (4/50 vs. 11/50). Lastly, there was a statistically significant difference in the 5 year survival between the HOPE group (94%) and the control group (78%), when censored for cancer recurrence.

### The Role of Potassium

One of the serious manifestations of reperfusion during liver transplantation is acute hyperkalemia, which can cause life-threatening arrhythmias. During organ reperfusion, the use of a preservation solution rich in potassium, graft cell lysis, massive transfusion of red blood cells as well as metabolic acidosis have been identified as contributing factors to increased potassium levels.

In 2018, Burlage et al. (26) examined potassium levels during DHOPE and SCS through both preclinical and clinical study. The preclinical analysis included 15 donor livers unsuitable for transplantation. Two groups were created: in the first one, six livers underwent 2 h of DHOPE followed by 6 h of *ex situ* NMP while, the second group consisted of nine livers that underwent 6 h of NMP without prior DHOPE perfusion. The data showed a negative correlation between the change in potassium levels upon *ex situ* reperfusion and the liver ATP levels after 2 h of NMP. On the contrary, there was a positive correlation between changes in sodium levels and ATP levels after 2 h of NMP. Moreover, high levels of potassium during *ex situ* reperfusion served as a strong predictor of peak ALT and lactate levels. The clinical study consisted of 10 recipients of livers that were DHOPE treated for 2 h prior to transplantation. The increase in potassium level during portal reperfusion showed a significant correlation with peak serum ALT levels post-transplantation. Interestingly, there was an increased need for noradrenaline in patients with increased potassium and decreased sodium levels. DHOPE treatment of the livers resulted in a decrease of potassium concentration in serum, which could be beneficial for patients with renal failure; a common comorbidity of patients with end-stage liver disease. For the same reason, DHOPE could be of benefit to patients with cardiovascular diseases given that potassium regulation plays a substantial role in stabilization of arrhythmias.

## NMP Clinical Trials and Studies

### First Successful NMP of Human Livers

In 2013, Op den Dries et al. (30) published the first series of successful NMP of human livers. They studied four discarded livers and monitored markers of hepatic function during NMP to assess their viability. ALT, GGT, and potassium concentrations remained stable. Results showed an increase followed by a decrease in lactate levels indicating an effective metabolic activity of the liver. The livers produced bile during the perfusion. Bile production gradually improved as levels of bilirubin and bicarbonate increased in bile throughout NMP. Lastly, there were no difference in hematoxylin and eosin (H&E) staining of liver biopsies taken before starting and at the end of the perfusion.

### Molecular Markers

Jassem et al. (41) matched 12 DBD NMP LTs to 27 DBD SCS LTs performed in the same period. They collected the perfusate at the end of NMP and as control, they perfused the SCS livers at the end of cold storage with 1 L of UW solution and collected it. They analyzed gene up-regulation in the two groups. Results showed up-regulation of 306 genes during NMP with the majority of them being related to tissue regeneration and platelet function. In contrast, there were 256 up-regulated in SCS with many of them being immune-related genes, such as proinflammatory cytokines and genes involved in neutrophil chemotaxis, humoral immunity, and platelet function.

They also assessed the impact of NMP on the proportion of hepatic mononuclear cells (HMCs) in perfusate compared to SCS. HMCs, that reflect the intrahepatic lymphocytes, are mainly represented by IFN-γ-producing liver-associated memory CD8+ T-cells. These cells are directly related to T cell activation and transplantation outcome (45). Their proportion of total cells and function are both affected by the mode of donor death, with DCD livers being less activated than DBD and living donors (45). The proportion of CD3-CD56+ NK cells tended to be higher in SCS than in NMP grafts (41). By contrast, the proportion of CD3+CD8+ T cells and CD4+CD25+FOXP3+ regulatory T cells were significantly higher in NMP than in SCS liver grafts. They then investigated whether there is an association between HMC function and the performance of the graft. Among HMCs collected after NMP, the length of donor intensive care unit (ICU) stay correlated positively with the frequency of IL-17-producing CD4+ T cells. In the setting of SCS, IL-10 producing CD4+ T cells correlated negatively with the duration of cold ischemia.

In the area of biomarker discovery, small non-coding RNAs, and more specifically microRNAs (miRs), are regarded as potential markers of cell function, stress, and injury.

In 2020, Matton et al. (44) investigated whether hepatocellular and cholangiocyte injury could be correlated with miRs in the perfusate and bile of NMP livers. Following a period of static cold storage, 12 livers underwent 6 h of NMP for viability assessment. In particular, they measured hepatocyte-derived miRs (HDmiRs) and cholangiocyte-derived miRs (CDmiRs) and correlated them to various parameters. Suzuki injury scores (46) calculated at the start and end of NMP were compared with their early predictors HDmiR-122, CDmiR-222, and their ratio in perfusate at 30 min. Scores prior to NMP start and after 6 h differed significantly; this is suggestive of progressive injury during NMP. They observed a significant positive correlation between the Suzuki injury score and miRs at the start of NMP. In addition, perfusate HDmiR122/CDmiR222 ratio after 30 min correlated positevely with the Suzuki injury score after 6 h of NMP. Furthermore, both HDmiR-122 and CDmiR-222 correlated positively with peak AST levels during the 6 h reperfusion period along with peak LDH in bile which correlated in a positive pattern with HDmiR-122. Lastly, there was a significant negative correlation between bilirubin levels in bile and early miR levels in perfusate at the end of NMP.

This study indicates that HDmiR-122 and CDmiR-222 measured in bile and perfusate early during NMP, could predict late function and injury markers. It is also important to mention, that the relative ratio of hepatocyte-derived and cholangiocyte-derived miRs could be used as a predictive marker of cumulative bile production during perfusion. Further studies should be conducted to examine HDmiR-122 and CDmiR-222 levels during various stages of liver transplantation and whether these markers could be used to accurately predict liver function post-operatively.

### NMP and Post Reperfusion Syndrome

In 2016, Ravikumar et al. (31) conducted the first series of liver transplantation after NMP. They performed 20 liver transplantations (16 DBD and 4 DCD) after NMP and matched those patients to 40 SCS livers (1 DBD and 1 DCD for each NMP; 1:2). There was a statistically significant difference in peak AST levels, with the most pronounced peak in the DCD cohort. Later that year, the same group published a retrospective study comparing 6 of the NMP-treated livers to 12 livers preserved with SCS and focused on post-reperfusion syndrome (PRS) in the two groups (32). Patients who received SCS-preserved livers exhibited signs of PRS and required high doses of vasopressors and blood products. On the other hand, recipients of NMP treated livers reached hemodynamic stability with lower doses of vasopressors and blood transfusions. These results are congruent with those of the Burlage et al. (26) pre-clinical study indicating that NMP is indeed capable of reducing proinflammatory cyotkines and hyperkalemia that increase the need for vasopressors during reperfusion.

Watson et al. (34) conducted a liver NMP transplant trial with focus on the effect of the level of oxygenation during perfusion. Twelve livers underwent NMP pretransplant, three DBD, and nine DCD. The first six were perfused under supraphysiologic (hyperoxic) conditions and the remaining six under normoxic conditions. Results showed that PRS, defined here as a reduction in mean arterial pressure (MAP) in the first 5 min of reperfusion to <70% of the baseline value during the anhepatic period, occurred in 5/6 patients who received hyperoxic livers compared to 0/6 in the normoxic group. In addition to this, vasoplegia, defined as a decrease in MAP during reperfusion to <50 mm Hg for more than 30 min, occurred in 4/6 in the hyperoxic group as opposed to 0/6 in the normoxic group. Peak ALT in the first 7 post-operative days was 1,210 vs. 780 in the hyperoxic and normoxic groups, respectively. Collectively, the data suggest that livers that underwent hyperoxic perfusion developed PRS possibly as a result of ROS release, whereas livers in the normoxic group had an uneventful reperfusion.

The same group published a NMP study with 47 livers. Twelve came from DBD donors and 35 from DCD donors (35). Twenty-two proceeded to transplantation of these, 12 have been described in the previous study. Their data indicated a correlation between the ALT concentration in the perfusate at 2 hrs and the peak ALT in the first 7 post-transplant days for the 22 transplanted livers, which is consistent with data from HMP studies.

### First Large Randomized Trial of NMP vs. SCS

In 2018, Nasralla et al. (33) published data from the first large randomized clinical trial conducted of NMP in liver transplantation. They successfully transplanted 121 livers treated with NMP and 101 preserved in cold storage. Their data show that PRS, here defined as more than 30% drop in mean arterial pressure persisting for more than 1 min within 5 min of reperfusion, was significantly more common in the SCS (32 out of 97) than the NMP group (15 out of 121). Only 10% of the NMP group patients developed EAD which was significantly lower than the EAD incidence in the SCS group (30%). The difference in the rate of non-anastomotic biliary stricture in the NMP group was not statistically significant. The lack of a statistical difference could be attributed to a number of factors including underpowering of the study for this outcome and the fact that there was no restriction on inclusion of high-quality livers for which there may be little benefit of perfusion.

### Logistical Issues of NMP

The logistics of continuous NMP can be challenging when organs have to be flown over great distances from a remote donor hospital, as some devices are not designed for transport in small aircraft. For those devices that are suited for air travel, providing perfusion over long distance transport is one of the great opportunities of NMP.

Ceresa et al. (37) looked into partially storing livers with SCS and then transition to NMP, thereby simplifying logistics and reducing costs. Thirty patients underwent liver transplantation using donor grafts subjected to post-static cold storage normothermic machine perfusion (pSCS-NMP), a technique sharing the same philosophy as HOPE in hypothermic machine perfusion. These patients were matched to 104 patients who received livers that underwent continuous NMP, part of the randomized clinical trial described before (33). A significant reduction in perfusate lactate and glucose was observed over the course of pSCS-NMP. Results also showed that livers in the pSCS-NMP study group had significantly lower lactate levels in perfusate and also significantly higher hepatic arterial flow rates at the beginning of preservation in comparison with the livers in continuous NMP comparator group. In addition, 10% of the pSCS-NMP treated livers developed PRS, while the same phenomenon was observed in 11% of the control livers. Although counterintuitive based on a large body of existing data show synergistic injury between the duration of warm and cold ischemia, these data are claimed to indicate that NMP after a period of SCS is both safe and feasible and could provide a solution to the logistical and cost drawbacks of continuous normothermic perfusion. However, a larger randomized study is needed in order to conclude NMP preceded by static cold storage as efficient and safe as continuous NMP.

Bral et al. (38) published a study in 2019, in which they focused on comparing livers procured at distant centers and transported in SCS for NMP at the recipient center (pSCS-NMP) to livers procured at recipient center with immediate initiation of NMP after procurement (local NMP). There were no significant differences in the characteristics of the recipients between the two groups. The primary outcome of 30-day patient and graft survival were 100% for both groups. The recipients of pSCS-NMP livers stayed for a shorter period of time in the intensive care unit (ICU) and got discharged earlier that the local NMP recipients. The 3 and 6-month patient survival rates were 100% for the recipients of the pSCS-NMP group and 88% for those of the local NMP group; a non-statistically significant difference. Although again a non-randomized experience, pSCS-NMP appears to be safe for low-risk donors. However, further studies including marginal livers are needed to reveal possible differences in outcomes when comparing back to base to local NMP.

Just this study was recently attempted in 2020 by Mergental et al. (43) in a nonrandomized phase 2 trial in which discarded DCD and DBD livers underwent NMP and viability assessment prior to transplantation. The livers were preserved under SCS for a median of >8 h during transfer to the transplant center where NMP was initiated. For a liver to be transplantable, it had to meet the major criterion of perfusate lactate level ≤2.5 mmol/L within 4 h of perfusion plus 2 of the following minor criteria; evidence of bile production, maintenance of perfusate pH ≥ 7.30, glucose metabolism, maintenance of arterial and portal flows (≥150 and ≥500 mL/min, respectively), and homogenous perfusion with soft consistency of the parenchyma. These criteria were established by this group in 2016 (47). One-hundred and eighty-five livers were offered for this study (43), 31 of which were included and perfused; 22 of these 31 livers met the viability criteria and were transplanted. Livers that met the viability criteria were removed from the NMP device during recipient hepatectomy, flushed with 3 L of histidine–tryptophan–ketoglutarate (HTK) solution at 4°C and immediately implanted. Results of these 22 liver transplantations were matched to 44 contemporaneous controls. There was no significant difference in patient and graft survival between the two groups in 1-year post-transplant. More importantly, there were both anastomotic and non-anastomotic biliary strictures in the study group, indication that SCS prior to NMP was unable to prevent biliary complications. This study highlights the deficiencies of the back to base approach for marginal organs; NMP initiation at the donor center could potentially help attenuate these preservation related complications.

### Clinical Criteria for Functional Assessment

Matton et al. (39) studied 23 human donor livers that were unsuitable for transplantation and used for research purposes. They focused on identifying biomarkers of bile duct injury (BDI) during NMP in a preclinical study and then apply those markers in a clinical trial. The preclinical study included 18 DCD and 5 DBD livers. They developed BDI scoring system ranging from 0 to 7. The score depends on degree of stromal necrosis, loss or injury of extramural peribiliary glands and necrosis of peribiliary vascular plexus. Comparison of livers with high BDI score to those with low score indicated that biliary pH, glucose, and bicarbonate concentrations were significantly higher in the low BDI score livers. Moreover, livers with high BDI score had two times higher biliary LDH concentration than those with low BDI score. Cholangiopathy is one of the major post-LT complications, especially for ECD livers, that are more prone to IRI (48). Therefore, developing a reliable method of predicting biliary function during perfusion could be critical.

Mergental et al. (36) aimed to develop a standardized NMP protocol, which would act as a tool of functional assessment of donor livers, as well as criteria predictive of liver viability. Twelve livers treated with NMP were divided into two groups based on lactate level. One group had an abrupt decrease in lactate levels that remained low throughout the perfusion, defined as the lactate-clearing (LC) group, whereas in the other group lactate levels fluctuated and eventually rose over time. This group was defined as the non-lactate-clearing (non-LC) group. After 6 h of NMP, livers of the LC group produced significantly more bile than livers of the non-LC group. Histologically, livers in the non-LC group presented small-droplet macrovesicular steatosis as well as apoptosis of biliary epithelial cells in the intrahepatic bile ducts. Lastly, microscopic analysis of the livers revealed flocculent densities, a sign of irreversible cell injury, in many mitochondria of the non-LC livers but not in those of the LC livers. Thus, trending early lactate clearance during NMP could provide insight on the viability of marginal organs going forward.

Ghinolfi et al. (40) conducted a randomized clinical trial including 20 transplanted patients, 10 of which received NMP livers and 10 received SCS livers. Perfusate analysis showed correlation between IL-6, IL-10, and TNF-a with lactate. Liver samples acquired 2 h post-reperfusion, revealed significant differences between the two groups. Results indicated that SCS preserved livers exhibited mitochondrial swelling, further lipid droplet accumulation, increase in dense bodies at the level of biliary poles (BPs), damaged endothelial cells, activated Kupffer cells and neutrophil infiltration. In contrast, examination of NMP livers revealed normal-size mitochondria, a reduced number of lipid droplets and only a few dense bodies in the BPs, whereas no damaged vessel endothelium or neutrophil infiltration was found. This study provided histological evidence that NMP is capable of reducing IRI. The observed correlation between TNF-α and perfusate lactate could be attributed to suboptimal livers or prolonged ischemia time, indicating the need for an improved NMP system that will purify the perfusate during perfusion and avoid or minimize the cold phase during procurement.

## Normothermic Regional Perfusion

*In situ* normothermic regional perfusion is used to restore perfusion of abdominal organs via cannulas in the aorta and vena cava using an extracorporeal circuit consisting of a membrane oxygenator, heater, and a pump. This can prevent ischemia of the liver and allows it to recover prior to SCS preservation and transportation.

Watson et al. (42) published data from 43 DCD liver transplants performed with 70 DCD livers that underwent normothermic regional perfusion (NRP). Reasons for not using the 27 livers on the trial included abnormal liver appearance [*n* = 7: steatosis (*n* = 3), fibrosis, cirrhosis, trauma, lesion], donor encephalitis of unknown cause (*n* = 1), bleeding (*n* = 1), abnormal liver function tests pre-NRP (*n* = 2), thromboembolism (*n* = 3), recipient unfit perioperatively (*n* = 1) rising ALT (*n* = 5, including 3 donors over 70 years old) and prolonged withdrawal period (*n* = 7). These livers were matched to 187 DCD liver transplants performed over the same period without NRP treatment. The incidence of early allograft dysfunction was significantly lower in the NRP group, mostly as a result of the significantly lower peak ALT in the first post-transplant week. They observed that non-NRP livers had more biliary complications. Interestingly, they noticed that none of the NRP treated livers developed cholangiopathy, whereas 27% of non-NRP livers actually did. Furthermore, donor age, recipient sodium concentration and locality of the liver predicted the development of ischemic cholangiopathy post-transplant.

## Combination of Three Machine Perfusion Techniques

Utilizing each method's advantages could be very beneficial. As already mentioned, single or dual hypothermic oxygenated machine perfusion ([D]HOPE) is capable of attenuating IRI. Controlled oxygenated rewarming (COR) serves as a gradual transition between cold preservation and warm reperfusion aiming to minimize organ damage. NMP mitigates ischemic injury and utilizes the metabolic activity of the organ at 37°C for functional assessment of the graft. However, extended static cold storage might interfere with the protective effect of NMP on IRI. An abrupt rewarming in the setting of depleted energy reserves may promote metabolic demands that outstrip available metabolic resources causing further injury. Combining DHOPE, COR, and NMP after a short period of SCS could accentuate the potential of MP.

Van Leeuwen et al. (27) conducted a prospective trial during which they studied 16 DCD livers that underwent DHOPE-COR-NMP after a period of SCS during transportation. Eleven of them were accepted for transplantation. The 5 rejected livers all had bile pH < 7.45. However, there was no statistically significant difference in graft and patient survival between recipients of DHOPE-COR-NMP livers and recipients of regular DBD and DCD livers without machine perfusion at 3, 6, and 12 months of follow up. Only 1 out of 11 livers developed post-transplant cholangiopathy at 4 months. Although that liver met all viability criteria, they retrospectively observed that the difference between the produced bile and perfusate pH, bicarbonate, and glucose would have been more indicative of biliary function than the absolute biliary pH value. These findings could help identify organs prone to poor post-transplant liver function during DHOPE.

## Future Applications of Machine Perfusion

Recently, He et al. (49) reported the first case of ischemia free transplantation of any vascularized organ in history of transplantation. Since the vascular supply to an organ must be disconnected from the donor and reconnected in the recipient, there is an obligatory period of ischemia and consequent reperfusion injury, albeit mild in some cases such as live donor transplantation when the ischemic period is minimized. In 2018, He et al. reported an innovative approach using machine perfusion to initiate flow in the donor before circulatory arrest. This was accomplished by cannulating the common bile duct for bile drainage, the infrahepatic inferior vena cava for outflow, the portal vein and hepatic artery for supply. After the *in situ* NMP circuit was established, the liver was harvested and moved to the organ reservoir under NMP. In the recipient, the liver is sewn-in while pump perfusion is still ongoing. In this manner, the liver is never without oxygenated blood flow and therefor never experiences ischemia and reperfusion injury. This exciting work is only relevant to brain death donation, but may relevant application in cases of high level hepatic steatosis causes and exaggerated ischemia-reperfusion injury in the recipient and limits use of many fatty livers (50).

There are a number of other innovative uses for MP that have the potential for clinical impact. Ongoing work suggests that steatotic livers may be de-fatted during *ex vivo* perfusion. Boteon et al. showed that pharmacological modulation of lipid metabolism during NMP can promote defatting of human steatotic livers within 6 h. This could lead to improved metabolic status of livers and their functional recovery and also reduce the reperfusion injury (51). This has been nicely demonstrated for steatotic Zucker rat livers and progress is being made with human livers. There are also a number of labs working on using machine perfusion to regenerate liver tissue *ex vivo*. If successful, this could render small portions of liver such as left lateral segments from deceased or live donors that are currently transplanted into pediatric recipients, also suitable for transplantation to adults. Finally, *ex vivo* maintenance of liver for days opens the door to potential genetic modification to modulate the immunogenicity or to target the inflammatory cascades associated with reperfusion of the graft.

## Conclusions

Machine perfusion is a disruptive technology with potential to have major impact on the safe and effective performance of liver transplantation. Both hypothermic and normothermic machine perfusion are capable of restoring and maintaining liver graft energy stores and mitigating ischemia-reperfusion injury post-transplant. Impact on other serious complications, such as ischemic cholangiopathy and PRS, that temper aggressive utilization of marginal organs awaits additional randomized trials. If successful, it is likely that MP will rapidly replace SCS as the standard mode of liver preservation. Alternative perfusion methods to NMP may help fill the logistical gap of NMP employment. HOPE remains a safe, easily used technique in order to increase pool of available organs by including marginal livers. While initiating NMP after a period of cold ischemia appears to be safe, it is insufficient to prevent ischemic cholangiopathy and PRS. In addition, NMP provides transplant teams with an extra important benefit of assessing liver function in real time and the ability to use a variety of markers to predict the quality and safety of the organ for transplantation. There are also novel interventions such as liver defatting, liver regeneration, and molecular and immunological modification that may be feasible under the physiologic conditions provided by NMP.

It seems clear that both HOPE and NMP methods are promising approaches in the field of organ preservation and further studies are needed in order to reach definitive conclusions about optimal approaches. As noted throughout this review, the vast majority of studies reported have been single center, non-randomized experiences. Additional large randomized clinical trials need to be conducted to fully define how to utilize machine perfusion of donor organs to secure recipients with the ultimate benefit. This is most vital for the marginal and currently discarded livers that hold the most promise for dramatic expansion of the pool of liver for transplantation. What seems certain despite the nascent nature of the technology, is that real-time monitoring and assessment of liver function through NMP is an asset in liver preservation and is from which tremendous additional potential remains to be unlocked.

## Author Contributions

NS, CR, and JM: study design and drafting of the manuscript. NS: sample and data acquisition. TC, DC, and RM: revised the manuscript. All authors reviewed the manuscript.

## Conflict of Interest

The authors declare that the research was conducted in the absence of any commercial or financial relationships that could be construed as a potential conflict of interest.
